# Modeling and prediction of cytotoxicity of artemisinin for treatment of the breast cancer by using artificial neural networks

**DOI:** 10.1186/2193-1801-2-340

**Published:** 2013-07-24

**Authors:** Abdolhossein Qaderi, Neda Dadgar, Hamidreza Mansouri, Seyed Ebrahim Alavi, Maedeh Koohi Moftakhari Esfahani, Azim Akbarzadeh

**Affiliations:** Department of Chemical Engineering, Science and Research Branch, Islamic Azad University, Tehran, Iran; Department of Agricultural Biotechnology, Science and Research Branch, Islamic Azad University, Tehran, Iran; Department of Pilot Nanobiotechnology, Pasteur Institute of Iran, Tehran, Iran

**Keywords:** Artemisinin, Cytotoxicity, Nanoliposome, Neural network

## Abstract

While artemisinin is known as anticancer medication with favorable remedial effects, its side effects must not be neglected. In order to reduce such side effects and increase artemisinin therapeutic index, nano technology has been considered as a new approach. Liposome preparation is supposed to be one of the new methods of drug delivery. To prepare the desired nanoliposome, certain proportions of phosphatidylcholine, cholesterol and artemisinin are mixed together. Besides, in order to achieve more stability, the formulation was pegylated by polyethylene glycol 2000 (PEG 2000). Mean diameter of nanoliposomes was determined by means of Zeta sizer. Encapsulation was calculated 96.02% in nanoliposomal and 91.62% in pegylated formulation. Compared to pegylated formulation, the percent of released drug in nanoliposomal formulation was more. In addition, this study reveals that cytotoxicity effect of pegylated nanoliposomal artemisinin was more than nanoliposomal artemisinin. Since artificial neural network shows high possibility of nonlinear modulation, it is used to predict cytotoxicity effect in this study, which can precisely indicate the cytotoxicity and IC50 of anticancer drugs.

## Introduction

While cancer is known to be one of the widespread diseases all around the world, breast cancer is considered to be the commonest type- 18% of all (Warner 2011; Vo and Millis 2012). Artemisinin, which is a terpene lactone taken from the leaves of Artemisia annua, is used as anti cancer drug (Yatuv et al. 2010; Lai and Singh 2006). Artemisinin molecule can damage the molecule and finally lead to the death of the cell (Lai and Singh 2006; Chen et al. 2009). However, what is not to be neglected is its side effects Chadwick et al. (2009). It seems practical to use nanocarriers in order to improve efficiency and paclitaxel toxic pattern. One of the controversial issues in nanotherapy concerns reduction or elimination of its side effects. Nanocarriers are used in nanotechnology as pioneer ones Peer et al. (2007). In this study liposomes are used as lipid nanocarriers Riaz (1996).

The Radial Basis Function (RBF) neural network methodology and the fuzzy means training strategy used for developing QSTR predictors of toxicity to *Vibrio fischeri* for a heterogeneous set of compounds (Georgia Melagraki et al. 2006). Industrial and municipal wastewaters constitute major sources of contamination of the aquatic compartment and represent a threat to aquatic life. Artificial neural networks based on three different learning paradigms were studied as a means of predicting acute toxicity to trout (Gagné and Blaise 1997). An artificial neural network in quantitative structure–activity relationship (QSAR) was developed for modeling of cytotoxicity data for anti-HIV 5-pheny-l-phenylamino-1*H*-imidazole derivatives. The obtained results show the validity of proposed model in the prediction of cytotoxity data of corresponding anti-HIV drugs (M. Arab Chamjangali et al. 2007).

Mathematical methods can be used to anticipate the amount of cytotoxicity and IC50 of either formulation. The aim of the study is liposome preparation and pegylation of artemisinin to gain improved therapeutic index, decrease side effects and also estimate of cytotoxicity and IC50 both of these formulations.

## Materials and methods

### Materials

Artemisinin, phosphatidylcholine, cholesterol, polyethylene glycol 2000 (PEG 2000) and MTT (0.5 mg/ml) resolution was purchased from Sigma Co. Besides, Invitrogen Co. Ethanol and isopropanol and RPMI 1640 medium were purchased from Merck Co. and Invitrogen Co., respectively. Also breast cancer cell line (MCF-7) was obtained from Pasteur Institute, Iran.

### Nanoliposomal and pegylated drug preparation

Cholesterol and phosphatidylcholine (ratio of 1 to 13.5) was dissolved in 100 ml of 98% ethanol (400 rpm, room temperature). Then 1 mg of artemisinin was added to the suspension and mixed by means of magnetic stirrer (300 rpm, 15 min, room temperature). After that, the solvent phase was evaporated by means of rotary evaporator (Heidolph Co., Germany) and the obtained gelos was dissolved in 12 ml of physiologic serum. In order to prepare pegylated nanoliposomal artemisinin some extra PEG 2000 was also added. Then the formulations are sonicated for 5 minutes (Bandelin Sonorex Digitec, 60 Hz).

### Size measurement of nanoliposomes

Nanoliposomes mean diameter was determined by Zeta sizer device (Malvern Instruments Ltd).

### Encapsulation efficiency

Equal volumes of prepared solutions were centrifuged (13000 rpm, 30 min, 4°C) in order to study the amount of paclitaxel encapsulated in both nanoliposomal and pegylated nanoliposomal formulations. Then, spectrophotometer (UV-1601 PC, Shimadzu Co.) was used to determine supernatant optical absorption of each formulation at wavelength of 195 nm. Formula 1 was used for calculation of the encapsulation (Zhang and Feng 2006).1

### Toxicity evaluation

After MCF-7 cellular culture, 100 μl of the suspension containing 10000 cells was poured into a 96-well plate and incubated (5% CO_2_, 37°C). Afterward, the supernatant was removed and different concentrations of nanoliposomal artemisinin formulation and its control, as well as pegylated nanoliposomal artemisinin formulation and its control, in addition to standard artemisinin was poured on cells; and then incubated for 24 hours on mentioned conditions. After that the supernatant was removed and 100 μl of MTT solution (0.5 mg/ml) was added. After 3 hours of incubation, purple (as to the formation of formazane) in was dissolved in live cells in 100 μl of isopropanol. Absorption was measured at wave length of 570 nm (spectrophotometer Power Eave XS) and IC50 was calculated by using Pharm program.

## Results and discussion

### Size measurement of nanoliposomes

Mean diameter of nanoliposomal artemisinin was measured 500 nm and that of pegylated nanoliposomal artemisinin was 455 nm which was considered to be in nano dimension size.

### Encapsulation efficiency

Encapsulation efficiency (EE %) was calculated through spectrophotometry method. Regarding encapsulation efficiency formulation, the amount of nonencapsulated artemisinin is obtained, which leads to determination of the amount of encapsulated drug, considering the amount of the initial drug. Encapsulation efficiency is 96.02% for nanoliposomal artemisinin formulation and 91.62% for pegylated nanoliposomal artemisinin formulation.

### Toxicity

MTT technique was hired to obtain toxicity. IC50 for nanoliposomal, pegylated nanoliposomal and standard drug is 2.1, 1.58 and 2.7 μl/ml by means of Pharm program.

### Artificial neural networks

Artificial neural networks are characterized by network architecture, node characteristics, and learning rules. The operation of neural networks is divided into two stages: learning (training) and generalization (recalling). Network training is typically accomplished by using examples, and network parameters are adapted using a learning method. This can be done in an online or offline manner. Once the network is trained to accomplish the desired performance, the learning process is terminated and it can then be used directly to replace the complex system dynamics. The trained network can be used to operate in a static manner: to simulate an unknown dynamics or nonlinear relationship Arbib (2002). Neural networks are usually biologically motivated. Each neuron is a computational node, which represents a nonlinear function. Most applications of neural networks utilize the function approximation capability of neural networks. These include modeling and system identification, regression and prediction. The neural networks are trained using supervised learning Feng and Ratnam (2000). The input and output layers are defined according to the dimensions of the input and output patterns. The input–output pattern pairs are fed to the network, and network parameters are adjusted accordingly. A trained network represents the learned nonlinear relation between the input–output pairs, and can generalize when an unknown input pattern is presented to the network Tipping (2001).

### Artificial neural network modeling

**Figure 1:**
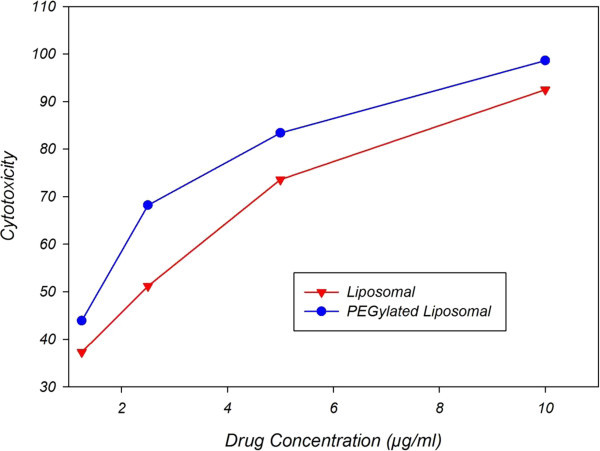
**Laboratory data for cytotoxicity of nanoliposomal artemisinin and pegylated nanoliposomal artemisinin for breast cancer treatment.**

In this study, cytotoxicity of artemisinin in breast cancer treatment by means of feed-forward artificial neural networks is modeled and predicted. Thus, a three-layer neural network is designed which incorporates 6 neurons in hidden layer and one in input layer for drug concentration and one neuron in output layer for cytotoxicity. Transfer function of Radbas is used for hidden layer neurons and Purlin transfer function is used for output layer; and Levevberg-marquardt backpropagation algorithm was used as network training procedure. Then, four sets of data including drug concentration and cytotoxicity are implemented to the network. After the training stage, two sets of new data, which have had no roles in network training, are implemented to the network as input according to Figure 1. And then, network output, including cytotoxicity, was anticipated.

**Figure 2:**
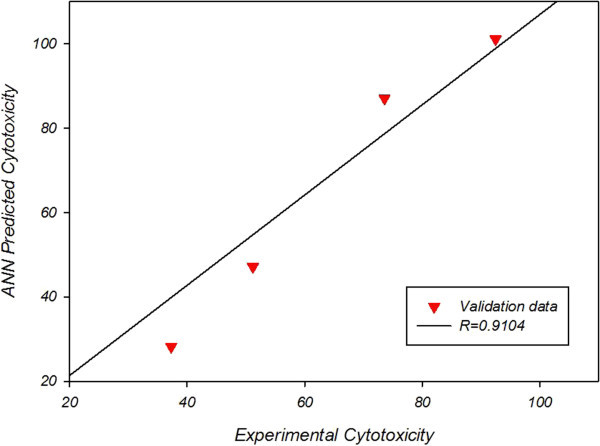
**Experimental vs predicted cytotoxicity for validation data of nanoliposomal drug.**

**Figure 3:**
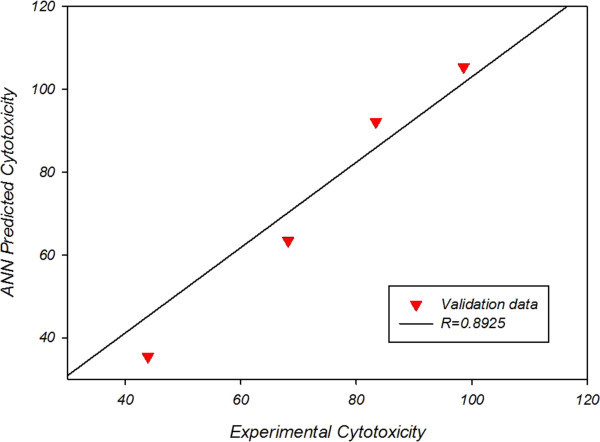
**Experimental vs predicted cytotoxicity for validation data of pegylated nanoliposomal drug.**

Figure 2 and Figure 3 reveal that neural network anticipation and experimental values for cytotoxicity are closely similar; in which network anticipation for cytotoxicity of nanoliposomal drug correlation of coefficient is R = 0.9104 with a mean square error of 29.3837 and that of pegylated nanoliposomal drug correlation of coefficient is R = 0.8925 with a mean square error of 37.0219.

## Conclusion

This study shows that neural network can provide an appropriate approach for drug cytotoxicity modeling and anticipation. This approach can be employed for cytotoxicity and IC50 anticipation of anti cancer drugs with very high accuracy.

## References

[CR1] Arab Chamjangali M, Beglari M, Bagherian G (2007). Prediction of cytotoxicity data (CC(50)) of anti-HIV 5-phenyl-1-phenylamino-1H-imidazole derivatives by artificial neural netcwork trained with Levenberg-Marquardt algorithm. J Mol Graph Model.

[CR2] Arbib M (2002). The handbook of brain theory and neural networks.

[CR3] Chadwick J, Mercer AE, Park BK, Cosstick R, O'Neill PM (2009). Synthesis and biological evaluation of extraordinarily potent C-10 carba artemisinin dimers against P. falciparum malaria parasites and HL-60 cancer cells. Bioorg Med Chem.

[CR4] Chen H, Sun B, Pan S, Jiang H, Sun X (2009). Dihydroartemisinin inhibits growth of pancreatic cancer cells in vitro and in vivo. Anticancer Drugs.

[CR5] Feng AS, Ratnam R (2000). Neural basis of hearing in real-world situations. Annu Rev Psychol.

[CR6] Gagné F, Blaise C (1997). Predicting the toxicity of complex mixtures using artificial neural networks. Chemosphere.

[CR7] Lai H, Singh NP (2006). Oral artemisinin prevents and delays the development of 7,12-dimethylbenz[a]anthracene (DMBA)-induced breast cancer in the rat. Cancer Lett.

[CR8] Melagraki G, Afantitis A, Sarimveis H, Igglessi-Markopoulou O, Supuran CT (2006). QSAR study on para-substituted aromatic sulfonamides as carbonic anhydrase II inhibitors using topological information indices. Bioorg Med Chem.

[CR9] Peer D, Karp JM, Hong S, Farokhzad OC, Margalit R, Langer R (2007). Nanocarriers as an emerging platform for cancer therapy. Nat Nanotechnol.

[CR10] Riaz M (1996). Liposomes preparation methods. Pak J Pharm Sci.

[CR11] Tipping M (2001). Sparse Bayesian learning and the relevance vector machine.

[CR12] Vo AT, Millis RM (2012). Epigenetics and breast cancers. Obstet Gynecol Int.

[CR13] Warner E (2011). Clinical practice. breast-cancer screening. N Engl J Med.

[CR14] Yatuv R, Robinson M, Dayan-Tarshish I, Baru M (2010). The use of PEGylated liposomes in the development of drug delivery applications for the treatment of hemophilia. Int J Nanomedicine.

[CR15] Zhang Z, Feng SS (2006). The drug encapsulation efficiency, in vitro drug release, cellular uptake and cytotoxicity of paclitaxel-loaded poly(lactide)-tocopheryl polyethylene glycol succinate nanoparticles. Biomaterials.

